# Prevalence of adverse events during ticagrelor versus clopidogrel treatment and its association with premature discontinuation of dual antiplatelet therapy in East Asian patients with acute coronary syndrome

**DOI:** 10.3389/fcvm.2022.1053867

**Published:** 2022-12-12

**Authors:** Min Gyu Kang, Jong Hwa Ahn, Kyehwan Kim, Jin-Sin Koh, Joeng Rang Park, Seok Jae Hwang, Yongwhi Park, Udaya S. Tantry, Paul A. Gurbel, Jin-Yong Hwang, Young-Hoon Jeong

**Affiliations:** ^1^Division of Cardiology, Department of Internal Medicine, Gyeongsang National University School of Medicine and Gyeongsang National University Hospital, Jinju, South Korea; ^2^Division of Cardiology, Department of Medicine, Irvine Medical Center, University of California, Irvine, Irvine, CA, United States; ^3^Department of Internal Medicine, Gyeongsang National University School of Medicine and Cardiovascular Center, Gyeongsang National University Changwon Hospital, Changwon, South Korea; ^4^Sinai Center for Thrombosis Research and Drug Development, Sinai Hospital of Baltimore, Baltimore, MD, United States; ^5^CAU Thrombosis and Biomarker Center, Chung-Ang University Gwangmyeong Hospital, Gwangmyeong, South Korea; ^6^Division of Cardiology, Department of Internal Medicine, Chung-Ang University College of Medicine, Seoul, South Korea

**Keywords:** acute coronary syndrome, ticagrelor, bleeding, dyspnea, adherence

## Abstract

**Background:**

Clinical evidence raises the issues regarding the high risk of adverse events and serious bleeding in East Asian patients receiving standard-dose ticagrelor treatment. We sought to evaluate the association between adverse events and their associations with premature discontinuation of dual antiplatelet therapy (DAPT).

**Methods:**

We enrolled East Asian patients presented with acute coronary syndrome who took DAPT with 90-mg ticagrelor (*n* = 270) or 75-mg clopidogrel (*n* = 674). During 1-month treatment, antiplatelet effect was evaluated with the VerifyNow P2Y12 assay, and the occurrence of Bleeding Academic Research Consortium (BARC) bleeding and modified Medical Research Council (mMRC) dyspnea was assessed with the dedicated questionnaire.

**Results:**

During 1-month follow-up, patients on ticagrelor showed the higher risks of bleeding (any BARC type: 45.6% vs. 23.6%; odds ratio [OR], 2.71 and BARC 1 or 2 type: 45.2% vs. 22.1%; OR, 2.90, respectively) and dyspnea (26.3% vs. 13.6%; OR, 2.25) compared with those on clopidogrel. In a receiver-operating characteristics curve analysis to predict bleeding risk, ticagrelor showed a lower cutoff of low platelet reactivity (LPR) (P2Y12 reaction unit [PRU] ≤ 20) than clopidogrel (PRU ≤ 110). Early occurrence of bleeding episode was significantly associated with LPR phenotype (OR, 2.68), not type of P2Y_12_ inhibitor. In multivariate analysis, type of P2Y_12_ inhibitor (ticagrelor vs. clopidogrel: OR, 2.19) and bleeding episode (OR, 2.94) were independent predictors for dyspnea occurrence. During 1-year follow-up, DAPT with ticagrelor showed a higher risk of premature discontinuation compared to DAPT with clopidogrel (27.8% vs. 4.7%; adjusted HR, 8.84), which risk appeared frequent during the first month (14.4%) during DAPT with ticagrelor. Early occurrence of bleeding and dyspnea synergistically increased a risk of DAPT non-adherence, irrespective of type of P2Y_12_ inhibitor.

**Conclusion:**

This analysis is the first evidence to show the different cutoff of low platelet reactivity during the reversible (ticagrelor) versus irreversible P2Y_12_ inhibitor (clopidogrel). Early occurrence of bleeding and dyspnea is very common during standard-dose ticagrelor treatment in East Asian patients, which show a close association with premature DAPT discontinuation.

**Clinical trial registration:**

[https://www.clinicaltrials.gov], identifier[NCT046 50529].

## Graphical Abstract

Central illustration. Association between type of P2Y12 inhibitor, adverse events and premature discontinuation of DAPT. After dual antiplatelet therapy (DAPT) with P2Y_12_ inhibitor plus aspirin, patients presented with acute coronary syndrome (ACS) were treated with percutaneous coronary intervention (PCI). Interestingly, the cutoffs of low platelet reactivity (LPR) related with bleeding episodes were different between reversibly (ticagrelor) vs. irreversibly binding P2Y_12_ receptor inhibitor (clopidogrel) (P2Y12 reaction [PRU] units measured by the VerifyNow P2Y12 assay: ≤ 20 vs. ≤ 110). However, prevalence of LPR was much higher during ticagrelor vs. clopidogrel treatment (68.1% vs. 16.5%). Dyspnea during 1-month DAPT with ticagrelor was associated with reversible binding to P2Y_12_ receptor by ticagrelor itself and non-P2Y_12_ receptor-mediated effect. Early occurrence (within 1 month) of bleeding and dyspnea appeared to have the synergistic impact on premature switch or discontinuation of DAPT. Finally, premature discontinuation of DAPT may be related with a worse clinical outcome after PCI. 
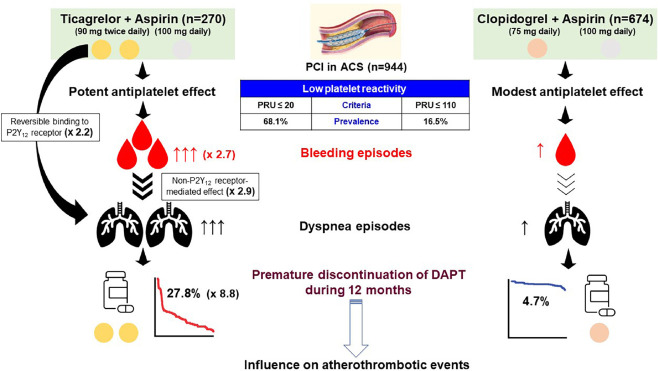

## Introduction

Although ticagrelor has been more effective in reducing atherothrombotic events than clopidogrel in patients with acute coronary syndrome (ACS) (1), there are emerging safety issues regarding bleeding episodes and dyspnea during ticagrelor treatment (2). Current data have challenged net clinical benefit of ticagrelor over clopidogrel in these patients (3). A large-scale retrospective cohort analysis has raised the concerns regarding increased risks of bleeding and dyspnea during ticagrelor versus clopidogrel treatment, which could lead to its early discontinuation (4). It was pointed out that the benefit of ticagrelor identified in a clinical trial may not be observed in a clinical practice (5).

Individualized approaches to weighting thrombotic and bleeding risk are required when selecting the potency and duration of antithrombotic strategy in ACS patients (6). Therefore, numerous clinical trials are evaluating the clinical benefit of de-escalation strategies, especially in cases of dual antiplatelet therapy (DAPT) including potent P2Y_12_ inhibitor. Clinical evidence is accumulating in terms with short-term DAPT by early aspirin discontinuation (7, 8), uniform reduced-dose strategy of potent P2Y_12_ inhibitor (9), or switching from potent P2Y_12_ inhibitor to clopidogrel (10–12).

Standard-dose ticagrelor (90 mg twice a day) is still western guideline-recommended antiplatelet regimen in high-risk ACS patients (6). The concept of ‘East Asian Paradox’ has been suggested by Dr. Jeong (13), which indicated the unique risk–benefit trade-off and pharmacokinetic profile of antithrombotic regimens in East Asian patients. In order to understand the conflicting results regarding clinical benefit of potent P2Y_12_ inhibitor vs. clopidogrel and prominent bleeding reduction with de-escalation strategy from East Asians (3, 4), further realistic researches are warranted to evaluate the linkage between potency/type of antiplatelet regimens and adverse events, and its contribution to drug adherence and clinical prognosis (14). We evaluated prevalence of bleeding and dyspnea during DAPT with ticagrelor versus clopidogrel, and their associations with its early switching or discontinuation of DAPT among East Asian patients presented with ACS.

## Materials and methods

### Study patients

**FIGURE 1:**
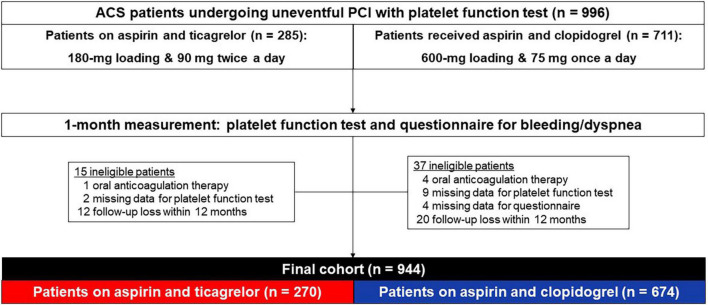
Flow diagram. ACS, acute coronary syndrome; PCI, percutaneous coronary intervention.

The study population was derived from the G-NUH (Gyeongsang-National University Hospitals, NCT04650529) registry, which was a prospective, two-center (Jinju and Changwon) database that enrolled percutaneous coronary intervention (PCI)-treated patients with coronary artery disease (CAD) (15). In this present data collected between September 2014 and December 2018, we evaluated the dedicated questionnaire-based prevalence of adverse events (e.g., bleeding, dyspnea) and platelet function measured by the VerifyNow test at 1-month follow-up in ACS patients receiving DAPT with ticagrelor versus clopidogrel. Patients were eligible for enrollment if they were hospitalized for an ACS, with or without ST-segment elevation, with an onset of symptoms during the previous 24 h. The diagnosis for ACS was defined by current guideline (6). The initial cohort consisted of PCI-treated patients with available on-admission VerifyNow test. Patients are excluded from this initial cohort when oral anticoagulant was administered, platelet function test or a questionnaire was missed at 1-month follow-up, or follow-up was lost within 12 months (Figure 1).

Baseline demographic, angiographic and procedural characteristics and clinical outcome data were collected prospectively. Patients were routinely followed at 1, 6, and 12 months after the index procedure and annually thereafter. Further information was collected by medical records or telephone contact if necessary. The institutional review board of the hospital approved the study protocol and waived the requirement for written informed consent for access to an institutional registry. The study was performed in accordance with the Good Clinical Practice Guidelines and the principles of the Declaration of Helsinki.

### Procedures and questionnaire survey

After arrival at the emergency room, suspected ACS patients were treated with standard loading doses of aspirin and a P2Y_12_ receptor inhibitor. Emergent PCI was performed after 300-mg aspirin plus 180-mg ticagrelor or 600-mg clopidogrel loading decided by the attending physician’s discretion. The enrolled patients were treated with timely PCI if subjects had significant coronary artery stenosis (>50% by visual estimate) and were eligible for PCI. For anticoagulation, unfractionated heparin was administered with dosage per label instructions (100 IU/kg). Glycoprotein IIb/IIIa inhibitors use was allowed during the procedure at the discretion of attending interventionists for bail-out cases (6). Following PCI, patients received ticagrelor 90 mg twice daily or clopidogrel 75 mg once daily in addition to aspirin 100 mg once daily. All patients were treated with the guideline-recommended pharmacological therapy (16).

After 1 month, adherence to pharmacologic therapy was assessed by meticulous interview, tablet counting, and dedicated questionnaire before outpatient treatment. In cases of earlier visit than expected, a questionnaire was acquired before an interview with a doctor. Questionnaire survey data were collected by a research coordinator. Bleeding episodes were determined by the questionnaire regarding the Bleeding Academic Research Consortium (BARC) criteria (17, 18). Dyspnea was categorized by using modified Medical Research Council (mMRC) scale (19) (Table 1). Classification of BARC bleeding type and mMRC dyspnea grade were performed by the main investigators (M. G. Kang and Y-H. Jeong).

**TABLE 1:** Definitions of bleeding and dyspnea episodes (17–19).

BARC bleeding criteria
**Type 1:** bleeding that is not actionable and does not cause the patient to seek unscheduled performance of studies, hospitalization, or treatment by a healthcare professional, which may include episodes leading to self-discontinuation of medical therapy by the patient without consulting a healthcare professional
**Type 2:** any overt, actionable sign of hemorrhage (e.g., more bleeding than would be expected for a clinical circumstance, including bleeding found by imaging alone) that does not fit the criteria for type 3, 4, or 5 but does meet at least one of the following criteria: (1) requiring non-surgical, medical intervention by a healthcare professional; (2) leading to hospitalization or increased level of care; (3) prompting evaluation.
**Type 3:** clinical, laboratory, and/or imaging evidence of bleeding with healthcare responses, as listed below: **Type 3a:** overt bleeding + hemoglobin drop of ≥3 to <5 g/dL or any transfusion with overt bleeding.
**Type 3b:** overt bleeding + hemoglobin drop of ≥ 5 g/dL, cardiac tamponade, bleeding requiring surgical intervention for control (excluding dental/nasal/skin/hemorrhoid), or bleeding requiring intravenous vasoactive agents. **Type 3c:** intracranial hemorrhage, intraocular bleeding compromising vision.
**Type 4:** CABG-related bleeding ● Perioperative intracranial bleeding within 48 h ● Reoperation following closure of sternotomy for the purpose of controlling bleeding ● Transfusion of ≥ 5 units of whole blood or packed red blood cells within a 48-hour period ● Chest tube output ≥ 2 L a 24-h period **Type 5:** fatal bleeding (bleeding directly causes death with no other explainable cause)
**Type 5a (probable):** no autopsy or imaging confirmation but clinically suspicious
**Type 5b (definite):** overt bleeding or autopsy or imaging confirmation
**mMRC dyspnea criteria**
**Grade 0:** breathlessness only on strenuous exercise
**Grade 1:** breathless when hurrying on the level or walking up a slight hill
**Grade 2:** walks slower than other people of same age on the level due to shortness of breath or need to stop for breath when walking at own pace
**Grade 3:** short of breath after walking few minutes on the level or about 100 yards (90 m)
**Grade 4:** Too breathless to leave the house, or breathless when dressing or undressing

BARC, Bleeding Academic Research Consortium; CABG, coronary artery bypass graft; mMRC, modified Medical Research Council.

### Platelet function test

Platelet function was evaluated with the VerifyNow P2Y12 assay before PCI and at 1-month follow-up. At admission, the blood sampling for platelet function testing (PFT) was taken before PCI, immediately after insertion of the arterial sheath in the catheterization laboratory. Time interval between antiplatelet loading and sampling was 2–12 h for non-ST-segment elevation ACS, whereas the sampling time after loading was relatively short (1–2 h) for ST-segment elevation ACS. During follow-up period, blood sampling was done 2–6 h after the last intake of the study medication from the antecubital vein. We encouraged to perform a follow-up PFT immediately after hospital arrival, before change in DAPT regimen when needs for change of P2Y_12_ inhibitor within 1 month.

Blood samples for platelet function analysis by VerifyNow assay (Accriva, San Diego, CA, USA) were collected in 3.2% citrate Vacuette tubes (Greiner Bio-One Vacuette North America, Inc., Monroe, NC, USA). This assay is a whole-blood, point-of-care, turbidimetric-based optical detection assay designed to measure platelet aggregation in response to an agonist that is based on the ability of activated platelets to bind to fibrinogen (20). The channel contains fibrinogen-coated polystyrene beads, 20 μM adenosine diphosphate, and 22 nM prostaglandin E1; the optical signal of this channel is reported as P2Y12 reaction units (PRU).

### Endpoints and outcomes

The primary endpoint was incidence of BARC bleeding and mMRC dyspnea reported by a dedicated questionnaire during 1-month ticagrelor or clopidogrel treatment. The secondary endpoints were: (1) determinants of bleeding and dyspnea during 1-month treatment; and (2) predictors of DAPT non-adherence during 12-month treatment.

During 1-year clinical follow-up, the data regarding major adverse cardiovascular events (MACE), BARC bleeding, and adherence to DAPT were collected at 1, 3, 6, and 12 months. MACE was defined as a composite of cardiac death, myocardial infarction (MI), or revascularization (21).

### Statistical analysis

The Kolmogorov–Smirnov test was performed to analyze the normal distribution of continuous variables. Continuous variables are presented as means ± standard deviation or as median (interquartile range [IQR]) as appropriate, while categorical variables are reported as frequencies and percentages. The Student unpaired *t*-test for parametric continuous variables and the Mann–Whitney *U* test for non-parametric continuous variables were used. Comparisons between categorical variables were performed using the Pearson Chi-square test or Fisher exact test, as appropriate. Receiver-operating characteristic (ROC) curve analysis was performed to find optimal cutoffs of continuous variables, which then were changed into the dichotomous covariates. All demographic characteristics and laboratory measurements were evaluated in univariate analysis for predicting the determinants of bleeding, dyspnea, and premature discontinuation of DAPT. Variables with *p*-value < 0.1 in the univariate analysis were then entered into the multivariate logistic regression analysis to provide odds ratio (OR) and 95% confidence interval (CI). The cumulative probability of adherence to DAPT and survival curves were constructed using the Kaplan–Meier method and compared using the log-rank test. A *p*-value < 0.05 was considered statistically significant, and statistical analyses were performed using SPSSv24.0 software (SPSS Inc., Chicago, IL, USA).

## Results

### Patient characteristics

A total of 944 patients with ACS (270 on DAPT with ticagrelor versus 674 on DAPT with clopidogrel) met the eligibility criteria (Table 2). Patients on DAPT (ticagrelor) were younger and had a higher incidence of dyslipidemia and smoking compared to those on DAPT (clopidogrel). Procedural characteristics and concomitant medications were comparable between the DAPT regimens.

**TABLE 2:** Baseline characteristics.

Variables	Ticagrelor	Clopidogrel	*P*-value
		
	(*n* = 270)	(*n* = 674)	
**Platelet function test (VerifyNow)**			
Before PCI, P2Y12 reaction units	138 ± 107	212 ± 84	<0.001
1-month follow-up, P2Y12 reaction units	22 ± 28	181 ± 69	<0.001
**Age, years**	61 ± 10	64 ± 12	<0.001
**Male, *n* (%)**	213 (78.9)	489 (72.6)	0.048
**Body mass index, kg/m^2^**	24.3 ± 3.1	23.9 ± 3.2	0.085
**Index presentation, *n* (%)**			<0.001
Unstable angina	21 (7.8)	100 (14.8)	
Non-ST-segment elevation myocardial infarction	163 (60.4)	295 (43.8)	
ST-segment elevation myocardial infarction	86 (31.9)	279 (41.4)	
**Risk factor or previous history, *n* (%)**			
Hypertension	113 (41.9)	338 (50.1)	0.021
Diabetes mellitus	58 (21.5)	198 (29.4)	0.015
Dyslipidemia	186 (68.9)	404 (59.9)	0.011
Smoking	121 (44.8)	251 (37.2)	0.033
Chronic kidney disease	59 (10.7)	99 (14.7)	0.116
Previous PCI	19 (7.0)	65 (9.6)	0.255
Previous ischemic stroke	8 (3.0)	43 (6.4)	0.038
**Laboratory measurements**			
White blood cell, x10^3^/mm^3^	10.5 ± 3.4	9.8 ± 3.7	0.011
Hemoglobin, g/dL	14.3 ± 1.5	13.6 ± 1.8	<0.001
Platelet, x10^3^/mm^3^	243 ± 71	246 ± 71	0.467
Glomerular filtration rate, mL/min/1.73 m^2^	87 ± 24	89 ± 29	0.357
Total cholesterol, mg/dL	200 ± 48	188 ± 46	<0.001
Hemoglobin A_1c_, %	6.2 ± 1.2	6.4 ± 1.3	0.036
Left ventricle ejection fraction, %	54 ± 9	54 ± 9	0.866
**Procedural characteristics**			
Culprit lesion			0.235
Left main coronary artery	1 (0.4)	6 (0.9)	
Left anterior descending artery	140 (51.9)	307 (45.5)	
Left circumflex artery	46 (17.0)	143 (21.2)	
Right coronary artery	83 (30.7)	218 (32.3)	
Multivessel disease, *n* (%)	108 (40.0)	359 (53.3)	<0.001
Multivessel PCI, *n* (%)	33 (16.6)	76 (12.5)	0.144
Treatment method			0.303
Drug-eluting stent	250 (92.6)	610 (90.5)	
Bioresorbable stent	2 (0.7)	3 (0.4)	
Bare-metal stent	0 (0)	4 (0.6)	
Drug-eluting balloon	3 (1.1)	20 (3.0)	
Balloon angioplasty	11 (4.1)	32 (4.7)	
Medical therapy	4 (1.5)	5 (0.7)	
Number of stent, *n*	1.2 ± 0.6	1.2 ± 0.7	0.735
Stent length, mm	33 ± 18	35 ± 20	0.180
**Concomitant medications, *n* (%)**			
Aspirin	270 (100.0)	666 (98.8)	0.114
Beta blocker	202 (74.8)	487 (72.3)	0.466
Angiotensin blockade	201 (74.4)	536 (79.5)	0.098
Statin	266 (98.5)	653 (96.9)	0.184

Continuous variables were expressed in mean ± SD or median (IQR) as indicated. PCI, percutaneous coronary intervention.

After antiplatelet loading, patients on DAPT (ticagrelor) showed a lower level of platelet reactivity than those on DAPT (clopidogrel) (138 ± 107 vs. 212 ± 84 PRU; △74 PRU; *p* < 0.001). After 1-month maintenance, ticagrelor treatment showed a markedly potent antiplatelet effect compared with clopidogrel treatment (22 ± 28 vs. 181 ± 69 PRU; △159 PRU; *p* < 0.001).

During 12-month follow-up, a total of four cases (1.5%) of MACEs (two cardiac deaths, one non-fatal MI, and one case of revascularization) occurred during ticagrelor treatment, whereas those receiving clopidogrel treatment showed 20 cases (3.0%) of MACEs (nine cardiac deaths, eight non-fatal MI, and four cases of revascularization) (*p* = 0.254).

### Incidence of bleeding, dyspnea and premature dual antiplatelet therapy discontinuation

The dedicated questionnaire-based prevalence of bleeding and dyspnea during 1 month was common irrespective of DAPT regimen (Table 3). Most of the events were reported as ‘BARC type 1 bleeding’ (86.2% of the total bleeding) and ‘mMRC grade 1 dyspnea’ (73.0% of the total dyspnea), respectively.

**TABLE 3:** Bleeding, dyspnea and premature discontinuation of initial dual antiplatelet therapy.

Variables	Ticagrelor	Clopidogrel	*P*-value
		
	(*n* = 270)	(*n* = 674)	
**Questionnaires at 1 month**			
**Any BARC bleeding**	**123 (45.6)**	**159 (23.6)**	**<0.001**
Type 1	111 (41.1)	132 (19.6)	<0.001
Type 2	146 (7.0)	23 (3.4)	0.015
Type 3	6 (2.2)	14 (2.1)	0.889
Type 4	0 (0)	0 (0)	1.000
Type 5	0 (0)	0 (0)	1.000
**Any mMRC dyspnea**	**71 (26.3)**	**92 (13.6)**	**<0.001**
Grade 1	54 (20.0)	65 (9.6)	<0.001
Grade 2	6 (2.2)	11 (1.6)	0.538
Grade 3	6 (2.2)	14 (2.1)	0.889
Grade 4	5 (1.9)	2 (0.3)	0.012
**Premature discontinuation of initial DAPT**			
**At 1 month**	39 (14.4)	5 (0.7)	<0.001
SAPT with aspirin	1 (0.4)	4 (0.6)	
SAPT with clopidogrel	1 (0.4)	1 (0.1)	
DAPT with aspirin plus clopidogrel	20 (7.4)	–	
Dose reduction, ticagrelor 60 mg twice daily	17 (6.2)	–	
**At 6 months**	59 (21.9)	11 (1.6)	<0.001
SAPT with aspirin	5 (1.8)	7 (1.0)	
SAPT with clopidogrel	1 (0.4)	3 (0.4)	
SAPT with prasugrel	–	1 (0.2)	
DAPT with aspirin plus clopidogrel	33 (12.2)	–	
Dose reduction, ticagrelor 60 mg twice daily	20 (7.4)	–	
**At 12 months**	75 (27.8)	32 (4.7)	<0.001
SAPT with aspirin	13 (4.8)	13 (1.9)	
SAPT with clopidogrel	5 (0.7)	17 (2.5)	
SAPT with prasugrel	–	1 (0.1)	
DAPT with aspirin plus clopidogrel	37 (13.7)	1 (0.1)	
Dose reduction, ticagrelor 60 mg twice daily	20 (7.4)	–	

BARC, Bleeding Academic Research Consortium; DAPT, dual antiplatelet therapy; mMRC, modified Medical Research Council, SAPT; single antiplatelet therapy.

During 1-month follow-up, bleeding episode was more frequently observed during ticagrelor versus clopidogrel treatment (45.6% vs. 23.6%; OR, 2.71; 95% CI, 2.01 to 3.65; *p* < 0.001). Furthermore, incidence of dyspnea was higher in patients receiving DAPT with ticagrelor versus clopidogrel (26.3% vs. 13.6%; OR, 2.25; 95% CI, 1.59–3.20; *p* < 0.001). However, prevalence of serious bleeding (BARC ≥ type 3 bleeding) and severe dyspnea (mMRC ≥ grade 3 dyspnea) was not different between the DAPT regimen (bleeding: 2.2% vs. 2.1%, *p* = 0.889 and dyspnea: 4.1% vs. 2.4%, *p* = 0.157, respectively).

**FIGURE 2:**
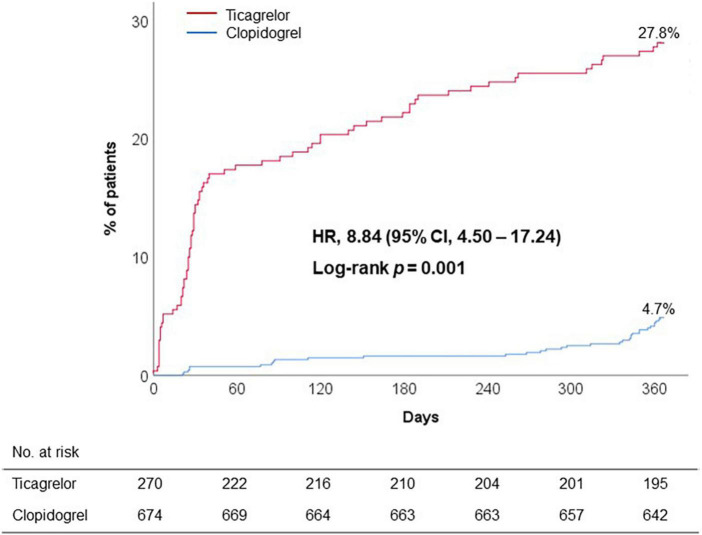
Premature discontinuation of initial DAPT regimen. CI, confidence interval; DAPT, dual antiplatelet therapy; HR, hazard ratio.

Discontinuation or switch of P2Y_12_ receptor inhibitor within 12 months was frequently observed during ticagrelor versus clopidogrel treatment (27.8% vs. 4.7%; adjusted OR, 8.84; 95% CI, 4.50 to 17.24; *p* < 0.001) (Figure 2). Majority of premature discontinuation of standard-dose ticagrelor occurred during 6 months (14.4% at 1 month and 21.9% at 6 months, respectively) (Table 3), in which switch to clopidogrel (12.2%) and reduced dose of ticagrelor (60 mg) (7.4%) were mostly applied to maintain the DAPT regimen.

### Determinants of adverse events and criteria of low platelet reactivity

**FIGURE 3:**
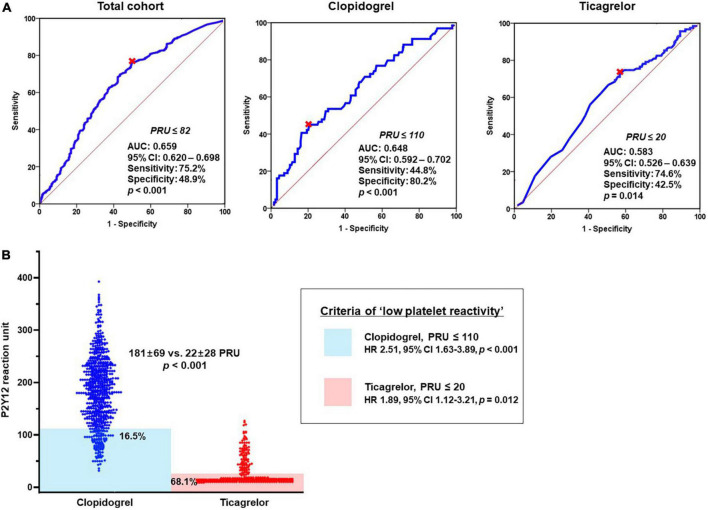
Association between bleeding episode and platelet reactivity according to DAPT regimen. **(A)** ROC curve analysis: the cutoffs of ‘low platelet reactivity’ and **(B)** Prevalence of ‘low platelet reactivity’ according to type of P2Y_12_ inhibitor. AUC, area under curve; CI, confidence interval; DAPT, dual antiplatelet therapy; OR, odds ratio; PRU, P2Y12 reaction unit; ROC, receiver-operating characteristics.

At first, we evaluated the association between bleeding episode and type of P2Y_12_ inhibitor. In a multivariable analysis, DAPT with ticagrelor was significantly associated with the increased risk of 1-month BARC bleeding compared to DAPT with clopidogrel (adjusted OR, 2.69; 95% CI, 1.97–3.67; *p* < 0.001) (Table 4A). ‘PRU ≤ 82’ was identified as the optimal cutoff of ‘low platelet reactivity (LPR)’ (with the greatest summation of sensitivity and specificity) to predict BARC bleeding at 1-month follow-up (area under curve [AUC], 0.659; 95% CI, 0.620–0.698; *p* < 0.001) (Figure 3A). For the next step, this LPR criteria was incorporated into the model for predicting bleeding (Table 4B). ‘PRU ≤ 82’ independently increased bleeding rate by about 2.7-fold (95% CI, 1.73–4.17; *p* < 0.001), which wiped out the impact of ticagrelor versus clopidogrel on bleeding occurrence. Therefore, increased risk of bleeding during ticagrelor versus clopidogrel treatment could be explained by its potent antiplatelet effect.

**TABLE 4:** Determinants of 1-month adverse events.

Variable	Univariable analysis			Multivariable analysis		
		
	OR	95% CI	*P*-value	OR	95% CI	*P*-value
**(A) Determinants of bleeding (BARC scale) during 1-month DAPT: model 1**						
Ticagrelor vs. clopidogrel	2.71	2.01–3.65	<0.001	2.69	1.97–3.67	<0.001
Age (per 1-year increase)	0.98	0.97–0.99	0.003	0.99	0.97–1.00	0.240
Body mass index (per 1-kg/m^2^ increase)	1.05	1.01–1.10	0.021	1.02	0.97–1.07	0.431
Index presentation with AMI	0.62	0.42–0.94	0.022	0.54	0.35–0.83	0.005
Hypertension	1.28	0.97–1.70	0.081	1.66	1.21–2.29	0.002
Diabetes mellitus	0.60	0.44–0.85	0.003	0.61	0.43–0.87	0.007
Smoking	1.31	0.99–1.74	0.061	1.28	0.92–1.79	0.135
Multivessel disease	0.63	0.48–0.84	0.001	0.75	0.55–1.01	0.061
**(B) Determinants of bleeding (BARC scale) during 1-month DAPT: model 2**						
Ticagrelor vs. clopidogrel	2.71	2.01–3.65	<0.001	1.56	0.83–2.04	0.240
Low platelet reactivity at 1 month*	3.21	2.40–4.31	<0.001	2.68	1.73–4.17	<0.001
Age (per 1-year increase)	0.98	0.97–0.99	0.003	0.99	0.97–1.00	0.375
Body mass index (per 1-kg/m^2^ increase)	1.05	1.01–1.10	0.021	1.02	0.97 – 1.07	0.363
Index presentation with AMI	0.62	0.42–0.94	0.022	0.52	0.34 – 0.81	0.004
Hypertension	1.28	0.97–1.70	0.081	1.71	1.24 – 2.37	0.001
Diabetes mellitus	0.60	0.44–0.85	0.003	0.63	0.44 – 0.91	0.014
Smoking	1.31	0.99–1.74	0.061	1.22	0.87 – 1.71	0.231
Multivessel disease	0.63	0.48–0.84	0.001	0.74	0.55 – 1.01	0.062
**(C) Determinants of dyspnea (mMRC scale) during 1-month DAPT**						
Ticagrelor vs. clopidogrel	2.25	1.59–3.20	<0.001	2.19	1.49–3.20	<0.001
Bleeding episodes	3.13	2.21–4.43	<0.001	2.94	2.02–4.25	<0.001
Age (per 1-year increase)	1.02	1.01–1.03	0.003	1.02	1.01–1.04	0.014
Female gender	1.53	1.09–2.25	0.017	1.13	0.74–1.73	0.544
Hypertension	1.61	1.05–2.28	0.006	1.22	0.83–1.80	0.298
Smoking	0.63	0.44–0.91	0.013	0.77	0.50–1.18	0.238
GFR (per 1-mL/min/1.73 m^2^ increase)	1.01	1.00–1.02	0.046	1.00	0.99–1.01	0.407
Previous ischemic stroke	2.55	1.39–4.70	0.002	1.89	0.96–3.71	0.064

**(B)** * ‘Low platelet reactivity’ (≤ 82 PRU measured by the VerifyNow test) was determined by ROC curve analysis for BARC bleeding episodes.

AMI, acute myocardial infarction; BARC, Bleeding Academic Research Consortium; CI, confidence interval; DAPT, dual antiplatelet therapy; GFR, glomerular filtration rate; mMRC, modified Medical Research Council; OR, odds ratio; PRU, P2Y12 reaction units; ROC, receiver-operating characteristics.

In addition, we evaluated the LPR cutoffs between ticagrelor versus clopidogrel (different property of binding to platelet P2Y_12_ receptor). For clopidogrel with irreversible binding property, ‘PRU ≤ 110’ was the optimal cutoff to predict bleeding episode (AUC, 0.648; 95% CI, 0.592–0.702; *p* < 0.001) (Figure 3A). In terms of ticagrelor with reversible binding property, the cutoff of LPR (‘PRU ≤ 20’) appeared lower compared with that of clopidogrel (AUC, 0.583; 95% CI, 0.526–0.639; *p* = 0.014). Patients who met the criteria of LPR were much greater during ticagrelor vs. clopidogrel treatment (68.1% vs. 16.5%; OR, 6.41; 95% CI, 4.70–8.74; *p* < 0.001) (Figure 3B). Irrespective of type of P2Y_12_ inhibitor LPR criteria was significantly associated with the risk of bleeding (clopidogrel: OR, 2.51; 95% CI, 1.63–3.89, *p* < 0.001 and ticagrelor: OR, 1.89; 95% CI, 1.12–3.21; *p* = 0.012, respectively).

In a multivariable analysis, occurrence of mMRC dyspnea during 1-month treatment was significantly associated with BARC bleeding episodes (OR, 2.94; 95% CI, 2.02–4.25; *p* < 0.001), as well as DAPT regimen (ticagrelor versus clopidogrel: OR, 2.19; 95% CI, 1.49–3.20; *p* < 0.001) (Table 4C), which supported that type of P2Y_12_ inhibitor may be related with dyspnea rate.

### Determinants of premature dual antiplatelet therapy discontinuation

**FIGURE 4:**
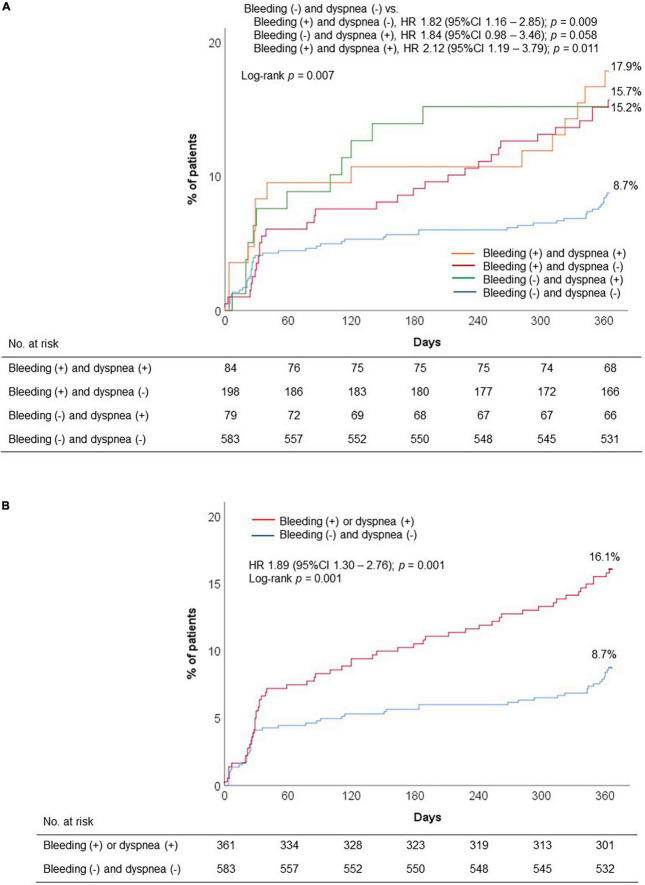
Premature discontinuation of DAPT according to early occurrence of adverse events: **(A)** Four groups: Presence of bleeding or dyspnea; and **(B)** two groups: presence of bleeding or dyspnea. DAPT, dual antiplatelet therapy; HR, hazard ratio.

Compared to DAPT with clopidogrel, DAPT with ticagrelor significantly increased the risk of discontinuation or switch of P2Y_12_ inhibitor (adjusted OR, 7.42; 95% CI, 4.76–11.55; *p* < 0.001) (Table 5A). In addition, we evaluated the impact of bleeding and dyspnea occurrence during 1 month on premature discontinuation of DAPT. Early occurrence of bleeding and dyspnea appeared to have the synergistic impact on premature discontinuation of DAPT, regardless of type of DAPT regimen (adjusted OR, 2.40; 95% CI, 1.23–4.54; *p* = 0.007) (Table 5B). The highest rate of premature DAPT discontinuation was observed in patients with both bleeding and dyspnea episodes (Figure 4A). In addition, early report of adverse events (bleeding or dyspnea during 1 month) was significantly associated with the risk of DAPT non-adherence (16.1% vs. 8.7%; HR, 1.89; 95% CI, 1.30–2.76; *p* = 0.001) (Figure 4B).

**TABLE 5:** Predictors for discontinuation or switch of DAPT regimen during 12 months.

Variable	Univariable analysis			Multivariable analysis		
		
	OR	95% CI	*P*-value	OR	95% CI	*P*-value
**(A) Predictors for discontinuation or switch of DAPT regimen: type of P2Y_12_ inhibitor**						
Ticagrelor vs. clopidogrel	7.63	4.90–11.76	<0.001	7.42	4.76–11.55	<0.001
Previous PCI	5.82	1.42–24.03	0.015	5.26	1.24–22.24	0.024
Previous ischemic stroke	3.33	0.80–13.91	0.098	2.25	0.51–9.81	0.279
LVEF (per 1-percent increase)	1.02	0.99–1.04	0.099	1.02	0.99–1.04	0.120

**(B) Predictors for discontinuation or switch of DAPT regimen: combined symptom**						
Previous PCI	5.82	1.42–24.03	0.015	5.85	1.42–24.37	0.015
Previous ischemic stroke	3.33	0.80–13.91	0.098	3.63	0.86–15.37	0.079
LVEF (per 1-percent increase)	1.02	0.99–1.04	0.099	1.01	0.99–1.04	0.163
Bleeding (–) and dyspnea (–) at 1 month vs.						
Bleeding (+) and dyspnea (–)	1.58	1.01–2.49	0.043	1.24	0.62–2.48	0.531
Bleeding (-) and dyspnea (+)	1.41	0.74–2.71	0.292	1.17	0.50–2.73	0.712
Bleeding (+) and dyspnea (+)	1.77	0.97–3.22	0.061	2.40	1.23–4.54	0.007

CI, confidence interval; DAPT, dual antiplatelet therapy; LVEF, left ventricle ejection fraction; OR, odds ratio; PCI, percutaneous coronary intervention.

## Discussion

This analysis is the first to evaluate the questionnaire-reported incidence of adverse events (bleeding and dyspnea) and its association with premature discontinuation of DAPT in East Asian patients presented with ACS. The main findings of the study are as follows (Central illustration): (1) A considerable proportion of patients suffered from early occurrence of bleeding or dyspnea (ticagrelor, 55.6% and clopidogrel, 31.3%); (2) early bleeding occurrence was significantly associated with antiplatelet effect, regardless of the type of P2Y_12_ inhibitor; (3) ticagrelor versus clopidogrel appeared to have different LPR cutoffs for bleeding occurrence (‘PRU ≤ 20’ vs. ‘PRU ≤ 110’); (4) early dyspnea occurrence was related with bleeding episodes, as well as the type of P2Y_12_ inhibitor (i.e., ticagrelor); and (5) early occurrence of bleeding and dyspnea synergistically increased the risk in premature discontinuation of DAPT, irrespective of the type of P2Y_12_ inhibitor.

Although ticagrelor is a potent non-thienopyridine and reversible-binding P2Y_12_ receptor inhibitor that has an overall positive clinical outcomes (1), clinicians encounter critical consideration regarding selection and maintenance owing to concerns of ticagrelor-induced bleeding, the sensation of dyspnea, and its adherence. Since our investigation was performed using questionnaire-reported adverse events (bleeding and dyspnea), the rates of bleeding and dyspnea during ticagrelor treatment appeared higher than the data from national registries (4). Similar to the PEGASUS-TIMI 54 (Prevention of Cardiovascular Events in Patients with Prior Heart Attack Using Ticagrelor Compared to Placebo on a Background of Aspirin-Thrombolysis in Myocardial Infarction 54) trial including patients with stabilized MI (24.1%) (2), the premature discontinuation of standard-dose ticagrelor in the present analysis appeared frequent (27.8%). Earlier meta-analysis showed the close relationship between adverse events and the risk of premature ticagrelor discontinuation (14). In line with the traditional concept, the present analysis also demonstrated that early occurrence of bleeding and dyspnea was a major determinant of premature discontinuation of DAPT in ACS patients. Therefore, the higher risk of bleeding, dyspnea and low adherence to DAPT observed in this cohort may be related to unique characteristics of real-world data including East Asian patients.

In the PLATO (Platelet Inhibition and Patient Outcomes) study, the rate of total major bleeding did not differ between ticagrelor versus clopidogrel treatment, but there was a significant difference in the risk of non-coronary artery bypass graft related major bleeding (1). In addition, Korean clinical data from the TICA-KOREA (Ticagrelor Versus Clopidogrel in Asian/Korean Patients with ACS Intended for Invasive Management) trial and the KAMIR-NIH (Korea Acute Myocardial Infarction Registry-National Institutes of Health) registry showed the significant increase of serious bleeding during ticagrelor versus clopidogrel treatment (by about twofold) (3, 22). These findings support the current concept of ‘East Asian Paradox,’ which indicates the different therapeutic range between high platelet reactivity (HPR) and LPR, and enhanced pharmacokinetic profile of ticagrelor/prasugrel in East Asian patients. It may be helpful to monitor the level of platelet reactivity to optimize antiplatelet maintenance regimen.

Our study firstly shows that ticagrelor versus clopidogrel have different LPR cutoffs (‘PRU ≤ 20’ vs. ‘PRU ≤ 110’), which may indicate a wider therapeutic window of reversible versus irreversible P2Y_12_ blockade. Preclinical data have suggested a lower level of platelet reactivity related with bleeding risk by reversible P2Y_12_ inhibitor (23). At the therapeutic doses, ticagrelor rapidly produces potent inhibition of adenosine diphosphate (ADP)-mediated platelet aggregation, with inhibitory activity correlating closely with plasma drug concentrations. In addition, ticagrelor binds to the P2Y_12_ receptor site at a site different from the ADP binding, is rapidly absorbed, and has a half-life of 7–12 h. Its reversibility of platelet binding may be associated with the more rapid offset of its platelet inhibitory effect, and consequently a wider separation between antithrombotic and bleeding effects than that seen with irreversible binding thienopyridines. The exposure of ticagrelor and its major active metabolite (AR-C124910XX) was also greater in East Asians as compared with Caucasians (13). For example, the exposure of ticagrelor and ARC124910XX was 40 and 48% higher in Japanese volunteers than in white volunteers after multiple doses of ticagrelor (100 mg twice daily). Although 90-mg ticagrelor has a low LPR cutoff compared with that of 75-mg clopidogrel in the present analysis, 68.1% of patients had a risk of LPR during ticagrelor treatment. Given the results, we suggest that the bleeding risk associated with the standard-dose ticagrelor in East Asian patients may be higher than in Caucasian patients. Therefore, a reduced-dose ticagrelor strategy would be challenging for East Asians (13).

The risk of bleeding and dyspnea during antiplatelet administration is associated with various causes. Compared with clopidogrel, ticagrelor has a reversible nature of P2Y_12_ inhibition at a non-ADP-binding site (24, 25). A plausible mechanism of ticagrelor-induced dyspnea would be increase of plasma adenosine level. Ticagrelor increases plasma adenosine level by inhibiting the sodium-independent nucleoside transporter-1, that can induce dyspnea by activating vagal C fibers on the bronchial wall (26). However, oral dipyridamole with a more potent adenosine uptake inhibitor than ticagrelor has not reported increased risk of dyspnea. Another possible mechanism of ticagrelor-induced dyspnea is its reversible binding with P2Y_12_ receptors in many cell lines including smooth muscle cells, neurons, and glial cells, which may suggest the relationship with a pattern of periodic breathing associated with increased chemosensitivity to hypercapnia. In addition, the present analysis provided a close association between bleeding and dyspnea. However, its mechanistic underpinnings warrant further investigations.

Our study suggests that dyspnea occurrence during ticagrelor treatment was related with bleeding episodes, and supports its class effect on adverse events (bleeding and dyspnea). Contrary to clopidogrel, the exposure of ticagrelor and its major active metabolite is greater in East Asians compared with Caucasians (13). Even for pharmacologic choice with clinically proven efficacy, maintaining adherence is essential (27). From the point of view, the risk of bleeding-related dyspnea might be higher in East Asians, which warrant a reduced-dose ticagrelor strategy to improve DAPT adherence (NCT04755387).

Although clopidogrel in combination with aspirin had been the choice of antiplatelet regimen to be used among patients with ACS (28), it has poor bioavailability and large inter-individual variability as compared with potent P2Y_12_ receptor inhibitor (29). If we apply the consensus-defined criteria of HPR (PRU > 208) for the present cohort, its prevalence was 55.9 and 0% during clopidogrel and ticagrelor treatment, respectively. Decreased response to clopidogrel in East Asian patients is related with a high carriage of the cytochrome P450 2C19 (CYP2C19) loss-of-function allele. For example, a large-scale PTRG-DES (Platelet Function and Genotype-Related Long-term Prognosis in Drug-Eluting Stent-treated Patients with Coronary Artery Disease) consortium including PCI-treated Koreans showed 47.9% of intermediate metabolizers and 14.2% of poor metabolizers, respectively (30). Therefore, standard-dose clopidogrel should be used cautiously in patients with high thrombotic risk.

### Study limitations

The present study was a retrospective analysis with a relatively small number of ACS patients, which may have a limitation for generalization. There were marked differences in baseline characteristics between the two groups, which may reflect the real-world practice and concern for selecting P2Y_12_ inhibitor in East Asian patients. However, we believe that statistical adjustment process may diminish the influence of this issue on determining clinical factors related with adverse events and DAPT discontinuation. Although use of DAPT including aspirin plus and P2Y_12_ inhibitor was recommended for the attending physicians, the limited cases of the clopidogrel group (1.2%) were discharged with P2Y_12_ inhibitor monotherapy, which was mostly related with allergic history or reaction to aspirin and a concern of bleeding (based on the electronic medical record and questionnaire survey). Temporal change in treatment modality can be an important confounder to consider, and these remained unadjusted. During antiplatelet treatment, daily risk of bleeding appears higher in the first month and decreases over time (31). Therefore, the linkage of bleeding with dyspnea occurrence and DAPT non-adherence would be changeable according to the disease phase. Finally, the sample size of the present analysis was underpowered to evaluate the association between the DAPT regimen and MACE occurrence and the observed risk of clinical events seemed lower than expected, which may be related to the inclusion criteria.

## Conclusion

In East Asian patients presented with ACS, early occurrence of bleeding and/or dyspnea is frequent (about 50% during 1 month) and the bleeding episode increases the risk of dyspnea during standard-dose ticagrelor treatment. The combined stratification by bleeding and dyspnea occurrence is significantly associated with the risk of premature DAPT discontinuation, which may suggest the unmet need to develop a de-escalation strategy with reduced-dose ticagrelor in these patients.

## Data availability statement

The raw data supporting the conclusions of this article will be made available by the authors, without undue reservation.

## Ethics statement

The studies involving human participants were reviewed and approved by Gyeongsang National University Hospital. Written informed consent for participation was not required for this study in accordance with the national legislation and the institutional requirements.

## Author contributions

MK and Y-HJ contributed to conception and design of the study. MK, JA, and Y-HJ organized the database. MK and Y-HJ performed the statistical analysis. MK wrote the first draft of the manuscript. All authors contributed to manuscript revision, read, and approved the submitted version.

## References

[1] Wallentin L Becker RC Budaj A Cannon CP Emanuelsson H Held C Ticagrelor versus clopidogrel in patients with acute coronary syndromes. *N Engl J Med.* (2009) 361:1045–57. 10.1056/NEJMoa0904327 19717846

[2] Bonaca MP Bhatt DL Oude Ophuis T Steg PG Storey R Cohen M Long-term tolerability of ticagrelor for the secondary prevention of major adverse cardiovascular events: a secondary analysis of the PEGASUS-TIMI 54 Trial. *JAMA Cardiol.* (2016) 1:425–32. 10.1001/jamacardio.2016.1017 27438319

[3] Park DW Kwon O Jang JS Yun SC Park H Kang DY Clinically significant bleeding with ticagrelor versus clopidogrel in Korean patients with acute coronary syndromes intended for invasive management: a randomized clinical trial. *Circulation.* (2019) 140:1865–77. 10.1161/CIRCULATIONAHA.119.041766 31553203

[4] You SC Rho Y Bikdeli B Kim J Siapos A Weaver J Association of ticagrelor vs clopidogrel with net adverse clinical events in patients with acute coronary syndrome undergoing percutaneous coronary intervention. *JAMA.* (2020) 324:1640–50. 10.1001/jama.2020.16167 33107944 PMC7592033

[5] Mullen L Meah MN Elamin A Aggarwal S Shahzad A Shaw M Risk of major bleeding with potent antiplatelet agents after an acute coronary event: a comparison of ticagrelor and clopidogrel in 5116 consecutive patients in clinical practice. *J Am Heart Assoc.* (2021) 10:e019467. 10.1161/JAHA.120.019467 33834845 PMC8174168

[6] Collet JP Thiele H Barbato E Barthélémy O Bauersachs J Bhatt DL 2020 ESC guidelines for the management of acute coronary syndromes in patients presenting without persistent ST-segment elevation. *Eur Heart J.* (2021) 42:1289–367. 10.1093/eurheartj/ehaa575 32860058

[7] Hahn JY Song YB Oh JH Chun WJ Park YH Jang WJ Effect of P2Y12 inhibitor monotherapy vs dual antiplatelet therapy on cardiovascular events in patients undergoing percutaneous coronary intervention: the SMART-CHOICE randomized clinical trial. *JAMA.* (2019) 321:2428–37. 10.1001/jama.2019.8146 31237645 PMC6593635

[8] Kim BK Hong SJ Cho YH Yun KH Kim YH Suh Y Effect of ticagrelor monotherapy vs ticagrelor with aspirin on major bleeding and cardiovascular events in patients with acute coronary syndrome: the TICO randomized clinical trial. *JAMA.* (2020) 323:2407–16. 10.1001/jama.2020.7580 32543684 PMC7298605

[9] Kim HS Kang J Hwang D Han JK Yang HM Kang HJ Prasugrel-based de-escalation of dual antiplatelet therapy after percutaneous coronary intervention in patients with acute coronary syndrome (HOST-REDUCE-POLYTECH-ACS): an open-label, multicentre, non-inferiority randomised trial. *Lancet.* (2020) 396:1079–89. 10.1016/S0140-6736(20)31791-8 32882163

[10] Angiolillo DJ Rollini F Storey RF Bhatt DL James S Schneider DJ International expert consensus on switching platelet P2Y12 receptor-inhibiting therapies. *Circulation.* (2017) 136:1955–75. 10.1161/CIRCULATIONAHA.117.031164 29084738

[11] Kim CJ Park MW Kim MC Choo EH Hwang BH Lee KY Unguided de-escalation from ticagrelor to clopidogrel in stabilised patients with acute myocardial infarction undergoing percutaneous coronary intervention (TALOS-AMI): an investigator-initiated, open-label, multicentre, non-inferiority, randomised trial. *Lancet.* (2021) 398:1305–16. 10.1016/S0140-6736(21)01445-8 34627490

[12] Cuisset T Deharo P Quilici J Johnson TW Deffarges S Bassez C Benefit of switching dual antiplatelet therapy after acute coronary syndrome: the TOPIC (timing of platelet inhibition after acute coronary syndrome) randomized study. *Eur Heart J.* (2017) 38:3070–8. 10.1093/eurheartj/ehx175 28510646

[13] Kim HK Tantry US Smith SC Jr Jeong MH Park SJ Kim MH The East Asian paradox: an updated position statement on the challenges to the current antithrombotic strategy in patients with cardiovascular disease. *Thromb Haemost.* (2021) 121:422–32. 10.1055/s-0040-1718729 33171520

[14] Arora S Shemisa K Vaduganathan M Qamar A Gupta A Garg SK Premature ticagrelor discontinuation in secondary prevention of atherosclerotic CVD: JACC review topic of the week. *J Am Coll Cardiol.* (2019) 73:2454–64. 10.1016/j.jacc.2019.03.470 31097167

[15] Bae JS Ahn JH Jang JY Cho SY Kang MG Kim KH The Impact of platelet-fibrin clot strength on occurrence and clinical outcomes of peripheral artery disease in patients with significant coronary artery disease. *J Thromb Thrombolysis.* (2020) 50:969–81. 10.1007/s11239-020-02103-w 32279217

[16] Dehmer GJ Badhwar V Bermudez EA Cleveland JC Jr Cohen MG D’Agostino RS 2020 AHA/ACC key data elements and definitions for coronary revascularization: a report of the American College of Cardiology/American Heart Association Task Force on clinical data standards (writing committee to develop clinical data standards for coronary revascularization). *Circ Cardiovasc Qual Outcomes.* (2020) 13:e000059. 10.1161/HCQ.0000000000000059 32202924

[17] Mehran R Rao SV Bhatt DL Gibson CM Caixeta A Eikelboom J Standardized bleeding definitions for cardiovascular clinical trials: a consensus report from the Bleeding Academic Research Consortium. *Circulation.* (2011) 123:2736–47. 10.1161/CIRCULATIONAHA.110.009449 21670242

[18] Jeong YH Oh JH Yoon HJ Park Y Suh J Lee SW Pharmacodynamic profile and prevalence of bleeding episode in East Asian patients with acute coronary syndromes treated with prasugrel standard-dose versus de-escalation strategy: a randomized A-MATCH trial. *Thromb Haemost.* (2021) 121:1376–86. 10.1055/a-1346-3300 33401330

[19] Mahler DA Wells CK. Evaluation of clinical methods for rating dyspnea. *Chest.* (1988) 93:580–6. 10.1378/chest.93.3.580 3342669

[20] Jeong YH Bliden KP Antonino MJ Park KS Tantry US Gurbel PA. Usefulness of the VerifyNow P2Y12 assay to evaluate the antiplatelet effects of ticagrelor and clopidogrel therapies. *Am Heart J.* (2012) 164:35–42. 10.1016/j.ahj.2012.03.022 22795280

[21] Garcia-Garcia HM McFadden EP Farb A Mehran R Stone GW Spertus J Standardized end point definitions for coronary intervention trials: the Academic Research Consortium-2 consensus document. *Circulation.* (2018) 137:2635–50. 10.1161/CIRCULATIONAHA.117.029289 29891620

[22] Kang J Han JK Ahn Y Chae SC Kim YJ Chae IH Third-generation P2Y12 inhibitors in East Asian acute myocardial infarction patients: a nationwide prospective multicentre study. *Thromb Haemost.* (2018) 118:591–600. 10.1055/s-0038-1626697 29534250

[23] Becker RC Gurbel PA. Platelet P2Y12 receptor antagonist pharmacokinetics and pharmacodynamics: a foundation for distinguishing mechanisms of bleeding and anticipated risk for platelet-directed therapies. *Thromb Haemost.* (2010) 103:535–44. 10.1160/TH09-07-0491 20135066

[24] Storey RF Bliden KP Patil SB Karunakaran A Ecob R Butler K Incidence of dyspnea and assessment of cardiac and pulmonary function in patients with stable coronary artery disease receiving ticagrelor, clopidogrel, or placebo in the ONSET/OFFSET study. *J Am Coll Cardiol.* (2010) 56:185–93. 10.1016/j.jacc.2010.01.062 20620737

[25] Becker RC Bassand JP Budaj A Wojdyla DM James SK Cornel JH Bleeding complications with the P2Y12 receptor antagonists clopidogrel and ticagrelor in the PLATelet inhibition and patient outcomes (PLATO) trial. *Eur Heart J.* (2011) 32:2933–44. 10.1093/eurheartj/ehr422 22090660

[26] Cattaneo M Schulz R Nylander S. Adenosine-mediated effects of ticagrelor: evidence and potential clinical relevance. *J Am Coll Cardiol.* (2014) 63:2503–9. 10.1016/j.jacc.2014.03.031 24768873

[27] Zeymer U Cully M Hochadel M. Adherence to dual antiplatelet therapy with ticagrelor in patients with acute coronary syndromes treated with percutaneous coronary intervention in real life. Results of the REAL-TICA registry. *Eur Heart J Cardiovasc Pharmacother.* (2018) 4:205–10. 10.1093/ehjcvp/pvy018 29878086

[28] Yusuf S Zhao F Mehta SR Chrolavicius S Tognoni G Fox KK. Effects of clopidogrel in addition to aspirin in patients with acute coronary syndromes without ST-segment elevation. *N Engl J Med.* (2001) 345:494–502. 10.1056/NEJMoa010746 11519503

[29] Stone GW Witzenbichler B Weisz G Rinaldi MJ Neumann FJ Metzger DC Platelet reactivity and clinical outcomes after coronary artery implantation of drug-eluting stents (ADAPT-DES): a prospective multicentre registry study. *Lancet.* (2013) 382:614–23. 10.1016/S0140-6736(13)61170-8 23890998

[30] Her AY Jeong YH Kim BK Joo HJ Chang K Park Y Platelet function and genotype after DES implantation in East Asian patients: rationale and characteristics of the PTRG-DES Consortium. *Yonsei Med J.* (2022) 63:413–21. 10.3349/ymj.2022.63.5.413 35512743 PMC9086699

[31] D’Ascenzo F Biolè C Raposeiras-Roubin S Gaido F Abu-Assi E Kinnaird T Average daily ischemic versus bleeding risk in patients with ACS undergoing PCI: insights from the BleeMACS and RENAMI registries. *Am Heart J.* (2020) 220:108–15. 10.1016/j.ahj.2019.10.001 31809991

